# Acute bullous hemorrhagic prurigo: a diagnostic challenge^⋆^^⋆⋆^

**DOI:** 10.1016/j.abd.2020.05.005

**Published:** 2020-09-18

**Authors:** Vidal Haddad Jr, Cecília Guimarães Ferreira Fonseca, Adriana Lúcia Mendes

**Affiliations:** a Department of Dermatology and Radiotherapy, Faculty of Medicine, Universidade Estadual Paulista, Botucatu, SP, Brazil; b Faculty of Medicine, Universidade Estadual Paulista, Botucatu, SP, Brazil; c Department of Internal Medicine, Faculty of Medicine, Universidade Estadual Paulista, SP, Brazil

**Keywords:** Anticoagulants, Diptera, Hypersensitivity, Insect bites and stings

## Abstract

Insect bites and bite wounds are quite common and most often have mild repercussions in humans. Statistics on the incidence of accidents caused by insects are not available, and the skin reactions after the bites are not always known. The authors present two cases of patients with hemorrhagic blisters on their hands after tabanidae bites and discuss the factors that cause the problem and the importance of the differential diagnosis of blisters with hemorrhagic content on human skin.

## Introduction

Insect bites and bite wounds are quite common and, in most cases, have mild repercussions on human skin. The statistics on the incidence of injuries caused by insects are not available, as most victims experience local reactions that are not reported.^1^ The risk factors for insect bites and stings are mainly related to environmental exposure, which occurs mainly in hot and humid places. Insect females bite to obtain hormonal maturation factors for their eggs and found it in the victims' blood.^1^

The most exposed people are children, people with large body exposure area, male individuals, people who have consumed alcohol, and pregnant women.^2^, ^3^ Some mosquitoes may also be drawn to the variation in the odor of sweat of menstruating women.^2^, ^3^ The regions of the body most affected are the extensor faces of the lower and upper limbs; bites on the palms, soles, and face are less common.^2^, ^3^

During the insect bite, potentially antigenic substances are introduced into human skin, which causes predisposed (especially atopic) individuals to experience exacerbated local inflammatory reactions. Another important fact is that flies and mosquitoes that suck human blood have anticoagulant substances in their saliva, which prevents obstruction of their digestive tract.^1^, ^2^, ^3^

Most bite reactions disappear spontaneously, and systemic allergic reactions that require emergency care are rare.^3^, ^4^, ^5^

The typical clinical picture of a bite from a sucking dipterus shows erythematous papules of varying sizes; the number of lesions is dependent on the victim's exposure. Itching is intense and results in abrasions and secondary infections.^4^

Immunocompromised individuals (people with HIV, cancer patients) may experience more severe local allergic reactions and some systemic signs, such as fever, malaise, headache, and lymphadenopathy.^1^ The stings persist for approximately two weeks, and progress to hypo- or hyperchromic macules that disappear after a few months.^2^

**Figure 1:**
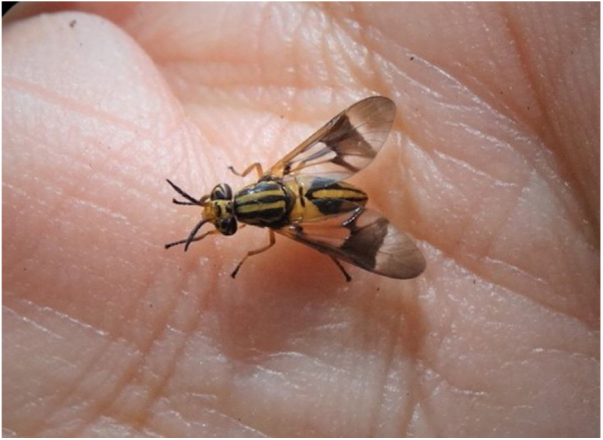
Horsefly or mosquito of the Tabanidae family, whose oral apparatus causes extensive lesions on human skin and can cause important allergic conditions. Photography: Vidal Haddad Junior.

If the patient is not atopic and sensitized, the symptoms of mosquito bites are mild. In turn, bites of black flies and horseflies cause local inflammation due to the fact that they are “chewers,” destroying the tissue and causing small hemorrhages at the bite sites. The horseflies of the Tabanidae family cause painful injuries, due to the insertion of the frontal stylus in the epidermis; their saliva contains allergens and pharmacologically active compounds that inhibit the body’s innate immune responses, causing anticoagulation, and impairing platelet formation, vasodilation, and anti-inflammatory processes (Fig. 1). In addition, horsefly stings can transmit infectious diseases caused by helminths, viruses, and bacteria, a fact that is little known but real, and obscured by painful stings and spoliation of blood, which harms the herds of horses and cattle.

## Case reports

### Patient 1

**Figure 2:**
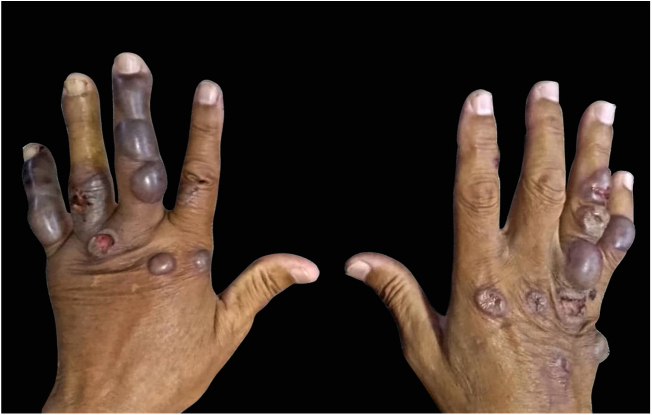
Blisters of hemorrhagic content on the hands of a patient who reported insect bites, but did not identify the aggressor. Note the extent of the lesions. Photography: Cecília Guimarães Ferreira Fonseca.

A 70-year-old retired fisherman from São Sebastião, state of São Paulo, who had arterial hypertension, and using losartan, hydrochlorothiazide, and amlodipine, was referred. He reported the appearance of a blister in his hand 15 days after an insect bite that he did not see; he denied the use of any substance or medication on the spot. He sought an emergency care unit; he informed he had been prescribed and was treated with antiallergic and benzathine penicillin, but new bullous lesions with hemorrhagic content were observed (Fig. 2). He denied pain, itching, or any other associated manifestation. In a new consultation, he was evaluated by a dermatologist, who recommended emptying of the blisters, as well as the use of prednisone, ciprofloxacin, sulfadiazine, and antiseptic soap, without improvement. The lesions disappeared after 15 days.

### Patient 2

**Figure 3:**
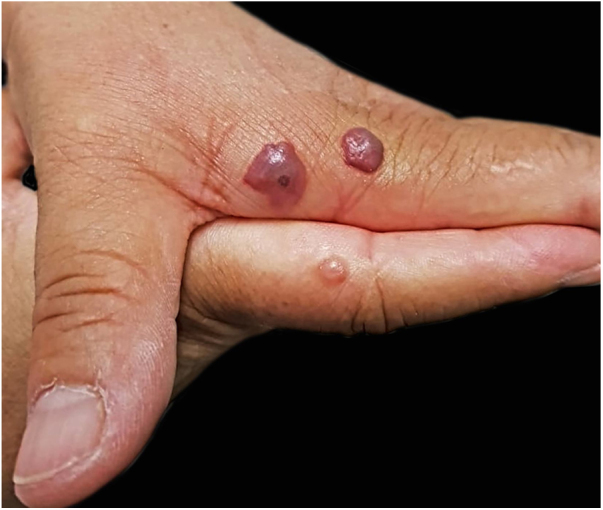
Blisters on the hands of a patient who identified the stings as being from horseflies. Some have hemorrhagic content and a central point where the bite occurred. Photography: Sílvia Mitiko Nishima.

**Figure 4:**
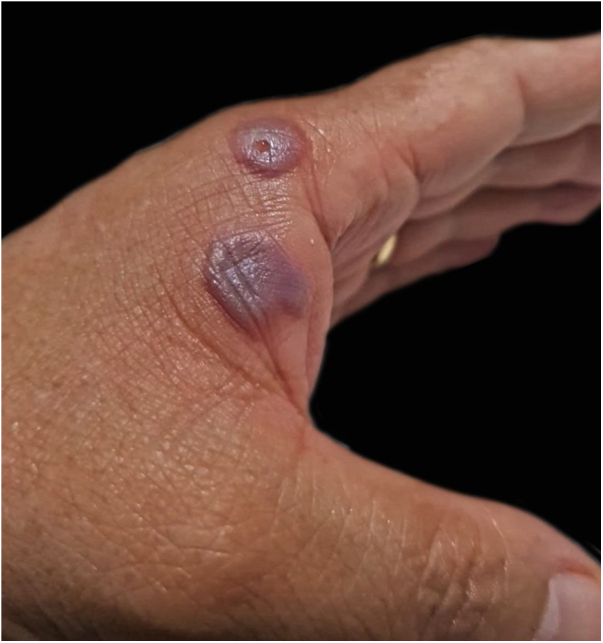
The blisters after one week, less tense, but still with the hemorrhagic content. Photography: Sílvia Mitiko Nishima.

A female patient, university professor of Biology, stung by horseflies during fieldwork – the insects were identified by the patient. The bites evolved into blisters with hemorrhagic content whose appearance made her seek help from a dermatologist. She presented blisters with hemorrhagic content of various diameters on her hands (Figure 3, Figure 4), which were treated with emptying of the liquid content and topical antibiotic in the resulting exulcerations, evolving with total cure in one week. The conditions of the location where the bites occurred and the absence of hospital care made it impossible to perform the histopathological examination, which would also be useful for the final diagnosis. The patient managed to collect the liquid from one blister, which was subsequently examined; cytology results demonstrated a large number of red blood cells.

## Discussion

Blisters and vesicles are not uncommon in bug bites. They appear in predisposed individuals, especially in atopic patients. The content of the lesions is usually citrine, reflecting the plasma present in the lesions. This is often observed in mosquito bites, whose sucking device is thin and introduces sensitizing substances into the victim. In the case of a simulid (black fly) or tabanid (horsefly) bite, the site is "chewed" by the sucking device, which severely injures the skin and causes minor bleeding, partly due to the hemorrhagic substances in the insects’ saliva. Tabanids (horseflies, mosquitoes) are flies measuring 6–33 mm, usually dark in color; they can also be yellowish. They have a large suction device, being more common in humid areas where cattle and equines, their main victims, are bred. The sting, which is very painful, causes bleeding and infections. If there is an overlapping allergic reaction, blisters are formed on the papules of acute prurigo, which may have hemorrhagic content due to the anticoagulant action of these insects’ saliva.^6^, ^7^ This can lead to diagnostic confusion with other causes of hemorrhagic blisters, such as autoimmune bullous diseases, polymorphic erythema, milker’s nodule, ORF, and other bullous diseases, which have a different clinical evolution. This occurs when the patient does not notice the bite or the insect which caused the lesion. Horseflies, which were associated with one of the described cases, are insects that can sensitize and cause hemorrhagic blisters on the hands of patients. Due to the similarity and atypical aspect of the lesions, these same insects may have also been implicated in the first case.

## Financial support

None declared.

## Authors’ contributions

Vidal Haddad Jr: Approval of the final version of the manuscript; conception and planning of the study, elaboration and writing of the manuscript, obtaining, analyzing, and interpreting the data; effective participation in research orientation, intellectual participation in propaedeutic and/or therapeutic conduct of studied cases; critical review of the literature, critical review of the manuscript.

Cecília Guimarães Ferreira Fonseca: Elaboration and writing of the manuscript, obtaining, analyzing, and interpreting the data; critical review of the literature.

Adriana Lúcia Mendes: Elaboration and writing of the manuscript; obtaining, analyzing, and interpreting the data; intellectual participation in propaedeutic and/or therapeutic conduct of studied cases, critical review of the manuscript, critical review of the literature.

## Conflicts of interest

None declared.
